# The role of genetic counsellors in genomic healthcare in the United Kingdom: a statement by the Association of Genetic Nurses and Counsellors

**DOI:** 10.1038/ejhg.2017.28

**Published:** 2017-03-22

**Authors:** Anna Middleton, Peter Marks, Anita Bruce, Liwsi K Protheroe-Davies, Cath King, Oonagh Claber, Catherine Houghton, Claire Giffney, Rhona Macleod, Claire Dolling, Sue Kenwrick, Diana Scotcher, Georgina Hall, Christine Patch, Laura Boyes

**Affiliations:** 1 Society and Ethics Research Group, Connecting Science, Wellcome Genome Campus, Cambridge, UK; 2 West Midlands Regional Clinical Genetics Service, Birmingham Women’s Hospital, Birmingham, UK; 3 North East Thames Regional Genetics Service, Great Ormond Street Hospital, London, UK; 4 All Wales Medical Genetics Service, University Hospital of Wales, Cardiff, UK; 5 Clinical Genetics Service, University Hospital Bristol NHS Foundation Trust, Bristol, UK; 6 Northern Genetics Service, Newcastle, UK; 7 Cheshire & Merseyside Regional Genetics Service, North West Coast Genomic Medicine Centre, Liverpool Women’s NHS Foundation Trust, Liverpool, UK; 8 Mater Misericordiae University Hospital, Dublin, Ireland; 9 Clinical Genetics, Cambridge University Hospitals NHS Trust, Cambridge, UK; 10 Manchester Centre for Genomic Medicine, St Mary’s Hospital, Central Manchester NHS Foundation Trust, and Division of Evolution and Genomic Sciences, School of Biological Sciences, Faculty of Biology, Medicine and Health, University of Manchester, Manchester Academic Health Science Centre, Manchester, UK; 11 Genomics England, Queen Mary University of London, London and Florence Nightingale Faculty of Nursing and Midwifery, King’s College London, London, UK

## Abstract

In the United Kingdom, genetic counsellors work together with clinical geneticists and clinical scientist colleagues within specialist genetics services, but they also often work in multidisciplinary teams (MDTs) outside of such services. There, they contribute genetic knowledge together with expert understanding of how to communicate genetic information effectively. They can offer education and support to the MDT, while providing management advice for both affected patients and the extended at-risk family members. As genomic technologies are implemented across many disciplines within healthcare, genetic counsellors are playing a key role in enabling non-genetic health professionals learn, understand and integrate genomic data into their practice. They are also involved in curriculum development, workforce planning, research, regulation and policy creation – all with the aim of ensuring a robust evidence base from which to practise, together with clear guidelines on what constitutes competence and good practice. The Association of Genetic Nurses and Counsellors (AGNC) in The United Kingdom (UK) and Republic of Ireland is committed to supporting genetic counsellors, across all sectors of healthcare and research, as they help deliver genomic medicine for the patient, family and world-class health services.

Genetic diagnosis, testing and counselling has occurred traditionally in a tertiary care setting within regional genetics centres (RGCs) comprising a MDT of clinical geneticists, genetic counsellors, and nurses and clinical scientists.^1^ Historically, clinical genetics focused on disorders showing Mendelian inheritance and on the wider implications of genetic diagnoses for the individual patient and their extended family.^1^ While this will continue to be important, RGCs are now responding to the unprecedented changes in genetic technology and the inevitable impact of genomic medicine across the whole of healthcare.^1^ Specialist genomic services are now available through RGCs in partnership with Genomic Medicine Centres (GMCs), hubs responsible for the delivery of the 100 000 Genomes Project in England. Health services in Scotland, Wales and Northern Ireland are also developing and implementing analogous genomics projects.^1^

Advances in technology promise to deliver the genomic data to inform healthcare on a mass scale and in a cost-effective manner. In the future, broad genomic testing in mainstream medicine settings, could enable more effective prediction of risk, management of disease and personalisation of treatment for both common and rare conditions. Developing this new technology with the required data handling, bioinformatics, interpretation, information, consent and clinical management of patients presents a significant challenge for the National Health Service (NHS). It is envisioned that genomic testing will increasingly be initiated outside the sphere of RGCs and will instead be integrated into a wide range of medical specialties. Realisation of the potential benefits will require a breadth of healthcare professionals to develop knowledge and proficiency in managing and communicating genomic information within their speciality.

Partnerships between RGCs and GMCs, and all other providers in healthcare will be critical in realising this vision of genomic medicine. Genetic healthcare professionals currently interpret and communicate complex genomic information while providing the support and counselling required for ‘whole person’ and ‘whole family’ care. As identified by the Human Genomics Strategy Group (2012), maintenance of this specialist workforce, both as a clinical service and as a resource to support integration of genomic technology and expertise within mainstream medicine, will be essential elements of the future genomics strategy.^2^ Historically, the delivery of genetics and genomics outside of specialist clinical genetics services has been variable.^3^ A combination of formal education programmes and continual reinforcement through clinical support will be required to deliver successful and sustainable training.^1^ Embedding the specialist expertise of the genetic counsellor in external MDTs is essential for achieving the enhanced, responsive education and training required for true integration of genomics in mainstream medicine.

## The role of the genetic counsellor

Genetic counsellors are internationally established as healthcare professionals with specialist education, training and assessed competence in genetics and genomics, combined with counselling skills.^4, 5^ Genetic counsellors are essential members of MDTs, enabling the genetic information generated to be appropriately relayed, to provide information and support to individuals and families attempting to comprehend and adjust to a genetic condition.^1^ Genetic counsellors have distinct skills, qualifications, experience and expertise to support individuals and families with genetic conditions by:^1^

**Figure 1:**
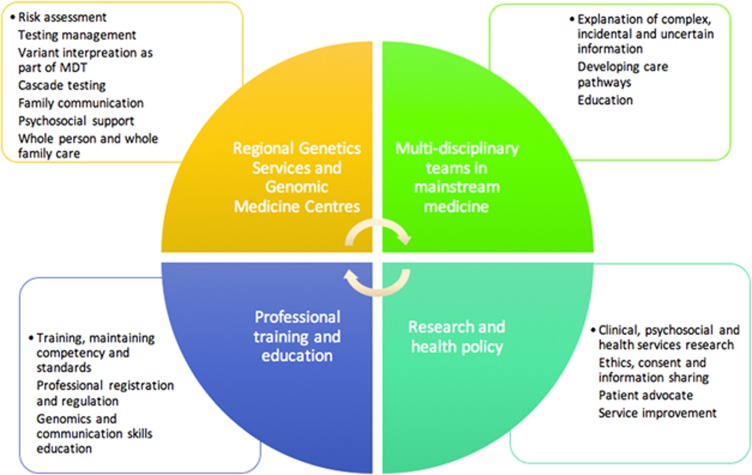
Genetic counsellor’s role in the implementation of genomics.

- Applying genomic information to overall future healthcare for an individual and family.
- Providing practical and psychosocial support for those with, and at risk from, genetic disease.
- Navigating the ethical challenges surrounding the disclosure and sharing of genetic information.
- Interpreting and explaining complex, incidental or uncertain genomic information.
- Providing education for the wider healthcare workforce on the clinical application of genomics. See Figure 1 for details.[Supplementary-material sup1]

**Figure 2:**
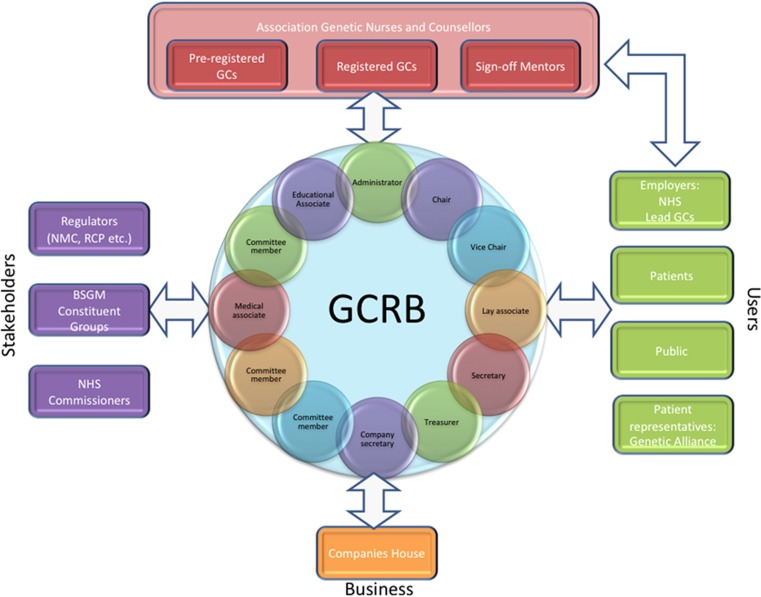
Gevernance structure for the Genetic Counsellor Registration Board (GCRB) UK and ROI.

The United Kingdom has been at the forefront of developing the profession and has established the gold standard for genetic counselling, which has been emulated across Europe and worldwide.^5, 6^ It is also highly valued by patients.^7^ In 2016, the Genetic Counsellor Registration Board (GCRB); see Figure 2 became accredited by the Professional Standards Authority (PSA); the PSA are an independent organisation, accountable to the UK Parliament, that offers voluntary registration for people working in health and social care.

Also in 2016, the first NHS Scientist Training Programme in genomic counselling began via the National School of Healthcare Science in England. Furthermore, it is anticipated that genetic counsellors will, for the first time, be included in UK national healthcare workforce planning with the aim of ensuring there are enough genetic counsellors being trained in the UK to meet the increasing demands of genomic healthcare.

Genetic counsellors in the United Kingdom and Republic of Ireland are embracing genomic technology and adapting their practise accordingly. They are a highly qualified and skilled workforce in a very strong position to make great contributions to the mainstreaming of genomics. The model of practice for genetic counsellors in the UK should be evaluated so that conclusions can be drawn about the relevance and reproducibility of this in mainland Europe and the rest of the world.
